# Ethical review key considerations for organoid applications in biomedical research—a systematic review

**DOI:** 10.3389/fcell.2026.1721946

**Published:** 2026-05-20

**Authors:** Guo Zhili, Zhang Nenghua

**Affiliations:** Jiaxing Hospital of Traditional Chinese Medicine, Jiaxing University, Medical Ethics Committee, Jiaxing, Zhejiang, China

**Keywords:** benefit-risk assessment, biomedical ethics, ethical review, organoid, stem cells

## Abstract

The organoid co-culture model, a novel tool for reconstructing three-dimensional microenvironments to study cell-cell interactions, has demonstrated significant potential in biomedical research in recent years. It holds considerable value for elucidating tumor immunosuppression mechanisms, conducting drug sensitivity tests, studying immune responses in infectious diseases, and revealing pathological features of neurodegenerative diseases. However, this model still faces challenges related to standardization, large-scale cultivation, and ethical regulation. The cellular sources of organoids—such as human pluripotent stem cells, adult stem cells, fetal tissues, or genetically edited cells—raise numerous ethical questions. These include: Is ongoing donor informed consent required? Does commercial use violate the original intent of donation? And do organoids possess attributes of “potential life”? Furthermore, the clinical translation of organoid technology continues to push ethical boundaries, necessitating appropriate governance frameworks. Each field of science and technology presents specific ethical questions regarding its application; risks, benefits, and monitoring procedures vary, thus requiring tailored review guidelines. This article aims to address how to resolve the ethical challenges associated with organoid technology and to clarify key considerations for ethical review.

## 1 Introduction

**FIGURE 1:**
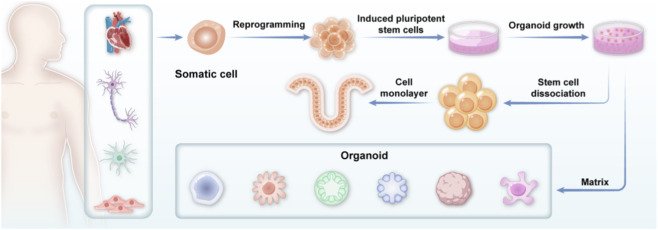
Schematic diagram of organoid research (the iPSC technology pathway centers on reprogramming somatic cells to a pluripotent state, which then serves as a foundation for directed differentiation into functional tissues. Key technical steps include stem cell dissociation for expansion and propagation via adherent monolayer culture).

Recent advances in stem cell technology, directed cell culture, and 3D tissue culture have propelled organoid research into an unprecedented stage of development. The fundamental approach involves using stem cells to construct three-dimensional tissue structures *in vitro*, simulating the architecture and function of human tissues and organs (Figure 1). The term “organoid co-culture model” specifically refers to an experimental system in which organoids of different origins, or organoids combined with other specific cell types or tissues, are co-cultured to simulate organ-organ interactions or disease microenvironments. Examples include co-culturing brain organoids with peripheral nerve organoids to study neural circuits, or tumor organoids with immune cells to mimic the tumor immune microenvironment.

Organoids have advanced rapidly in areas such as human development modeling, precision medicine, regenerative medicine, drug screening, and disease modeling, promising transformative impacts on biomedicine (Rossi et al., 2018). The fidelity of organoids has improved significantly, with scientists successfully cultivating models resembling the human brain, liver, intestine, pancreas, prostate, kidney, retina, and other organs (Jaconi and Puceat, 2021). Notably, many countries now permit the use of organoids and other alternative methods in preclinical drug testing to reduce reliance on animal experiments (Park et al., 2024). This regulatory shift is expected to further accelerate progress in the field.

However, because organoid research utilizes human cells to simulate human organs—including the brain—in vitro, and given the technology’s early stage and inherent uncertainties, it raises complex and nuanced ethical controversies. These include specialized concerns such as the procurement and use of brain organoids, and the moral status of embryo-like structures. Consequently, this article seeks to explore how to navigate the ethical challenges posed by organoid technology and to identify strategies for promoting responsible and sustainable innovation.

### 1.1 The application value of organoids

In disease modeling, organoids have successfully simulated the development of various complex diseases, proving particularly valuable for research on genetic disorders, infectious diseases, and cancer. They provide a platform that more closely approximates human physiology compared to traditional animal models or two-dimensional cell cultures (Fontanelli et al., 2025).

Organoid technology has become a pivotal tool in biomedical research due to its remarkable ability to mimic human organ structure and function. Yet its rapid advancement—especially its demonstrated potential in disease modeling, drug screening, and regenerative medicine—profoundly challenges established ethical and regulatory frameworks. The “quasi-life” attributes of organoids, particularly brain organoids, along with their potential for sentience and their reliance on complex human biomaterials, raise questions that existing ethical guidelines are not fully equipped to address (Koplin and Savulescu, 2019).

Presently, there is a lack of systematic guiding principles for the ethical review of organoid research. Existing frameworks often struggle to effectively evaluate its unique risks, such as the potential emergence of consciousness, the delineation of moral status, informed consent for source materials, and the governance of translational research. There is, therefore, an urgent need for a more targeted ethical review system to steer responsible innovation (Wan et al., 2023).

This study aims to address this gap by proposing a systematic ethical review framework. Centered on eight core dimensions, the framework is designed to provide researchers, ethics committees, and policymakers with clear assessment benchmarks, thereby helping to safeguard ethical and humanistic values while supporting scientific progress.

### 1.2 Key ethical review considerations in organoid research

If organoid research is regarded as an extension of stem cell research, then organoid ethics should similarly be viewed as an extension of stem cell ethics. Given that organoids are derived from stem cells, stem cell ethics should form the baseline for organoid ethics, encompassing core issues such as: cell procurement, responsible research conduct, and the management of donor and public expectations (Munsie and Gyngell, 2018).

Stem cell procurement raises distinct ethical issues depending on the cell source and type of biological material used. To date, much debate has centered on embryo collection. The use of surplus *in vitro* fertilized (IVF) embryos for research has sparked public discourse concerning the moral status of embryos and the permissibility of their use as research material (O’Connell and Winter, 2020). The procurement of oocytes is similarly contentious, involving concerns about donor compensation, potential exploitation, and undue inducement. The reprogramming of stem cells obtained from consenting donors into induced pluripotent stem cells (iPSCs) introduces questions regarding donor information and consent. Although established informed consent procedures exist, the unique nature of organoids may necessitate updates to these protocols and a reconsideration of their implications (Marei, 2025).

Furthermore, issues such as the legality of organoid sources, ethical boundaries of use, data and privacy protection, bioethical controversies, regulatory compliance, and benefit-sharing require careful deliberation. These points will be elaborated in the following sections (Table 1).

**TABLE 1:** Summary of key ethical review dimensions for organoid research.

No.	Ethics review dimension	Core focus	Specific considerations	Clear definition	Current practices/Guidelines	Key ethical dilemmas	Practical guidelines for ethics review committees
1	Legitimacy of sources	Compliance in biomaterial acquisition	① Specialized informed consent from donors (exclusively for organoid research). ② Traceable tissue sources (with filing through legal hospitals/biobanks). ③ Embryonic stem cells must adhere to international/national embryo research guidelines. ④ Formal approval procedures for biobank establishment	The original biomaterials (e.g., somatic cells, stem cells, tissue samples) used for organoid generation must be obtained through legal, compliant, and ethical channels.	Currently, there are no specialized ethical protocols for organoids; most rely on existing biobank guidelines. Regulations on embryonic stem cell use vary significantly across countries, with no unified implementation standards.	Can “broad consent” cover unforeseen future organoid research? How to define the boundaries for the secondary use of discarded clinical samples?	Establish a dynamic review mechanism to ensure donors autonomously authorize the use of their biomaterials for specific organoid research and related activities after full informed understanding.
2	Informed consent	Protection of donor autonomy	① Consent forms must specify organoid usage, storage duration, and scope of secondary research. ② Donors have the right to withdraw consent at any time. ③ Disclosure of commercialization potential and benefit-sharing mechanisms	After fully understanding research details (including purposes, risks, and benefits), donors autonomously authorize the use of their biomaterials and may withdraw consent at any time—with ongoing dynamic follow-up throughout the process.	Most consent forms are overly broad and fail to cover organoid-specific complexities, such as long-term culture, gene editing, potential sentience, and chimera applications.	How to balance the specificity of consent with research unpredictability? Are donors entitled to share intellectual property or commercial benefits derived from organoids, and if so, in what form?	Reject research proposals with vague or overly broad consent forms; require researchers to establish a phased re-consent mechanism (e.g., re-obtaining consent when shifting from disease modeling to drug screening); rigorously review the fairness and feasibility of benefit-sharing plans.
3	Scope of use	Research restrictions and prohibitions	① Prohibition on implantation into human/animal embryos. ② Ban on creating neural organoids with human-like sentience (specialized review required for brain organoids). ③ Prohibition on germline gene editing	Define technical redlines and application boundaries for organoid research to prevent ethical slippery slopes and unacceptable moral risks.	International consensus prohibits human germline editing and uterine implantation of organoids. However, assessing the complexity of brain organoids and setting limits on neural integration in animal chimeras remain exploratory areas without unified quantitative standards.	How to define and detect the thresholds for “sentience” or “consciousness” in brain organoids? To what stage should the development of human-animal chimeras be permitted?	Initiate specialized ethical reviews for brain organoid or high-integration chimera research, involving experts in neuroscience and ethics; require researchers to submit detailed monitoring plans and pre-defined experimental termination criteria.
4	Data and privacy	Protection of donor information	① Anonymization of data. ② De-identified storage of genetic information. ③ Compliance with data protection regulations such as GDPR and HIPAA	Protect donors’ sensitive information (e.g., genomic, phenotypic data) to prevent identification, leakage, or misuse.	Follows general biomedical data protection regulations. However, the risk of re-identification increases with interconnected organoid databases, and multi-omics data integration poses additional challenges to privacy protection.	How to balance the research value of organoid data with privacy protection? Will strict privacy safeguards hinder data sharing?	When reviewing data management plans, assess the risk of donor re-identification from organoid data; require the establishment of a tiered data access system and clarify compliant solutions for cross-border data transfer.
5	Ethical controversy response	Addressing the “quasi-life” attribute	① Avoid anthropomorphic naming (e.g., “mini-brain”). ② Ethical protocols for organoid disposal (including those with neural activity). ③ Respect for cultural/religious definitions of the origin of life	Address ethical disputes regarding the moral status and degree of respect owed to organoids (especially brain and heart organoids) due to their structural and functional complexity.	There are no universally recognized norms for organoid disposal. Some institutions have developed internal guidelines, but cultural differences impede the formation of consensus.	Should organoids exhibiting brain-like electrical activity be granted certain “subject rights”? What disposal methods (e.g., simple discard, ritualized handling) constitute “respect”?	Require researchers to avoid misleading terms such as “mini-brain”; evaluate the ethical appropriateness of experimental endpoints and disposal plans for neural organoids, and consult external ethics experts when necessary.
6	Animal ethics	Ethics of chimera research	① Integration of human cells may induce “human-like consciousness”. ② Moral ambiguity of chimeras. ③ Disclosure of chimera applications in informed consent forms	Evaluate the ethical challenges posed by human-animal chimera research (involving implantation of human organoids/cells into animals) to animal welfare, human dignity, and species boundaries.	Regulations vary globally, with strict restrictions on embryonic chimeras and constraints on neural integration in adult animals (e.g., the brain). Chimera applications are rarely mentioned in donor consent forms.	At what level of human cell proportion or integration in animals does an ethical crisis arise? Will this blur the fundamental boundary between humans and animals?	Scrutinize the scientific necessity of chimera research; require detailed methods and plans for monitoring human cell integration levels; ensure informed consent forms explicitly disclose the potential use of donors’ biomaterials in chimera creation.
7	Benefit sharing	Fair distribution of commercialization benefits	① patents must acknowledge donors’ contributions. ② A portion of profits to fund public health initiatives. ③ Prevention of “biopiracy”	Ensure that benefits from organoid research, especially commercialization gains, are fairly shared with society, the research community, and donor populations.	Donors’ contributions are often unrecognized and rarely rewarded with tangible benefits. While some ethical guidelines recommend benefit-sharing with source communities, these provisions are generally non-binding and lack concrete enforcement frameworks.	Should donors be regarded as “contributors” or “collaborators”? In what form (monetary rewards, healthcare benefits, recognition, etc.) should benefits be shared?	Review intellectual property and commercial interest clauses in research agreements; encourage or require researchers to submit a preliminary benefit-sharing plan (even a framework); enhance fairness reviews for research involving vulnerable populations or rare disease groups.
8	Oversight and compliance management	Implementation of multi-level regulation	① initial review by the institutional review board (IRB). ② Filing with national health and technology authorities (for research involving human embryos or sensitive genes). ③ International collaborations must adhere to the declaration of Helsinki and bilateral regulations	Establish a clear, cohesive multi-level oversight and compliance system (from institutional to national/international levels) to ensure the responsible conduct of organoid research.	Regulation lags behind technological development, with emerging technologies often falling into gray areas of existing frameworks. Conflicts in standards are common in international collaborations.	Should regulation be technology-based or application-based? How to harmonize global standards to avoid “ethics dumping”?	Clarify the committee’s role and responsibilities within the multi-level regulatory system; proactively communicate with national regulatory authorities for high-risk research; for international projects, adhere to the “highest standard” ethical principle and ensure compliance with regulations of all participating parties.

## 2 Methods

**FIGURE 2:**
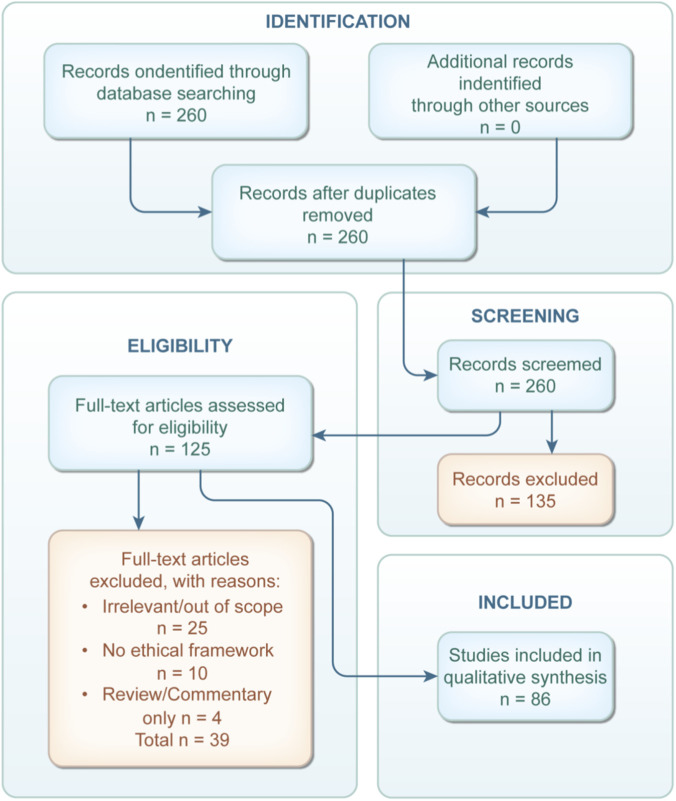
PRISMA flow diagram of included literature.

Unlike systematic reviews in empirical disciplines, there are currently no established guidelines or manuals for conducting systematic literature reviews on bioethical topics, though some recommendations have been published. Where applicable, PRISMA guidelines (Figure 2) were consulted. The review protocol has not been published or registered.

### 2.1 Search and selection strategy

A literature search was conducted in June 2025 across five bioethics and biomedical databases: PubMed, EMBASE, Medline, and Web of Science Core Collection. Supplemental systematic searches were performed using Google Scholar for specific documents. Search terms were developed based on keywords, their truncations, and relevant database-specific subject headings pertaining to organoids and key ethical review points. Only articles in English were considered for full-text analysis.

We screened all titles and abstracts on the topic published before June 2025, with no restrictions on publication date. At this stage, articles meeting the predefined inclusion/exclusion criteria were selected based on their title and abstract. Any discrepancies regarding eligibility were resolved through discussion to reach a consensus prior to full-text retrieval. Following title and abstract screening, full texts were assessed. Finally, the reference lists of selected full-text articles were examined to identify any potentially missed scientific articles or documents, which were included if they fully met the criteria.

### 2.2 Inclusion and exclusion criteria

All articles that mentioned and described ethical issues, questions, or challenges related to organoids were included. Biomedical articles focusing solely on organoid technology development without addressing any ethical considerations were excluded.

### 2.3 Analysis and synthesis

A qualitative content analysis approach was adopted. This inductive method was used to categorize ethical issues. The analysis was structured around the main theme of “key ethical review points for organoids in research applications,” with sub-themes including organoid technology principles, discussions of eight ethical dimensions (e.g., informed consent, risk-benefit assessment, privacy, dignity), and special topics such as brain organoids and chimeras.

### 2.4 Quality appraisal

Due to the lack of suitable or applicable criteria for assessing the quality of the included literature, no formal quality assessment procedure was performed. This is a well-documented limitation in systematic reviews of ethics literature. Instead, the depth of discussion concerning the identified ethical themes within each included paper was evaluated.

## 3 Results

A PRISMA flow diagram illustrating the selection process was created. The database search yielded 260 records. Based on title and abstract screening, 125 were deemed eligible. Following reference checking and full-text screening, 86 articles were ultimately included for analysis.

### 3.1 Legitimacy of sources

Once established, organoid cell culture models create a sustained demand for primary cell line sources. Cultivating patient-derived primary organoids requires approval from ethics committees and informed patient consent, a process that can involve considerable time for prospective enrollment (Hendriks et al., 2021). Given that organoids carry significant societal impact and substantial economic value with commercial potential, a primary ethical challenge lies in ensuring equitable benefit distribution among all stakeholders while guaranteeing the legitimacy of these sources (Maier et al., 2021).

Although organoid culture technology emerged nearly 15 years ago, its translation into clinical and pre-clinical applications remains hindered by non-standardized procedures and ambiguous protocols (Presley et al., 2022). There is an urgent need to establish comprehensive guidelines and unified protocols, including stringent standardized control systems to verify the legitimacy of cell line sources. Organoid biobanks present specific ethical and practical challenges, necessitating a paradigm shift within both scientific and societal frameworks of responsible research and innovation. This shift must involve a reconsideration of consent procedures, commercial access and commodification, as well as privacy and ownership issues (Hong et al., 2023).

As collaborative partners and participants, stakeholders are crucial for organoid biobank research aimed at translational and precision medicine. A primary concern is that, at the time of consent for biobank inclusion, the specific nature of future research remains unknown. Therefore, implementing models of dynamic consent and participatory governance over time becomes essential. This research paradigm aims to foster responsible biobanking by establishing and maintaining ongoing relationships between biobanks and participants, particularly within complex tissue biobanks designed for translational medicine (Li H. et al., 2022).

Biobanks should be reconceptualized as collaborative processes that extend beyond the act of donation. This involves establishing transparent, inclusive, participatory, and reciprocal agreements among all relevant actors. Within such a collaborative ecosystem, each participant serves as an active and irreplaceable component, contributing to sustainable development through principles of mutual reciprocity. Such biobanks are essential for developing innovative scientific models that are responsive to evolving data requirements (Taglieri et al., 2025).

### 3.2 Informed consent

Implementing effective informed consent procedures for the use of human biological materials and information constitutes a primary challenge in the application of this technology. This process is decisive in safeguarding the fundamental rights and interests of donors or patients who provide biological materials (Neziri et al., 2024). Within the informed consent framework, donors must be comprehensively and clearly informed about how their biological materials will be used in scientific research or clinical practice, ensuring they possess a complete understanding and retain autonomous decision-making rights. This is not only a matter of respecting the dignity of the donors but also a fundamental requirement of research ethics (Hoppe et al., 2023).

The dynamic consent model is a digitally-based, continuous, and interactive framework for informed consent. Designed to empower donors of biological samples or data, it enables them to receive ongoing updates on project progress, new findings, and potential future uses throughout the research lifecycle via secure IT platforms—such as dedicated patient portals or mobile applications. Donors can also provide or withdraw real-time, specific authorization for each new research use of their samples. Implementation typically involves establishing an initial tiered-consent structure within organoid biobanks or long-term research programs, providing donors with regular updates through the platform, and triggering specific re-authorization requests if research diverges into new directions beyond the original consent scope. For example, using established organoids for unanticipated disease studies would require such re-authorization. This approach transforms dynamic consent into an actionable tool to address ethical challenges arising from the longevity and inherent uncertainty of organoid research.

Donor informed consent is a prerequisite for research involving donated biological materials. For existing cell lines or archived materials, even when donors have provided broad initial consent, neither party may have anticipated the subsequent high likelihood of successful organ replication *in vitro* (Parente et al., 2024). In such cases, ensuring that consent remains truly informed—given future biotechnological uncertainties which might otherwise preclude researchers from constructing new biotechnological entities from archived materials or conducting tests like genome sequencing—necessitates further management through dynamic consent mechanisms (Bredenoord et al., 2017).

When substantial advances occur in cell culture techniques, broad initial consent may become insufficient, and “dynamic consent” is required. Informing donors that their cells can develop into organoids can be challenging but is essential. This includes clarifying potential misunderstandings about the nature of organoids (which can be described as *in vitro* “mini-organs”) and addressing therapeutic misconceptions (where patients may expect direct clinical benefits, while most research remains basic or preclinical, contributing to general knowledge rather than personalized medicine) (Barnhart and Dierickx, 2022). Additionally, sufficient information must be provided regarding potential future uses of derived products, such as their transfer between laboratories and countries, integration with other biotechnologies, and possible commercialization. Donors should be aware that donating to a biobank is a future-oriented and potentially irreversible commitment (Tao et al., 2025).

Other issues concerning the donor relationship include data anonymization and the handling of potential incidental findings. For instance, if organoids, serving as organ models, make complete anonymization more difficult, or if incidental findings become more frequent and precise, the level of protection afforded to donors will need to be correspondingly enhanced (Thakar and Fenton, 2023).

### 3.3 Scope of use

Ethical literature on the clinical application of organoid technology primarily focuses on two areas: personalized medicine (*in vitro*) and the transplantation of organoids into the human body (*in vivo*).

#### 3.3.1 Organoids in personalized medicine

Patient-derived organoids can be used for drug testing (Zhou et al., 2024), thereby enabling personalized treatment strategies. As *in vitro* models of individual patients, organoids can help predict drug efficacy for that specific person. Consequently, they are viewed as a novel form of evidence to inform clinical decision-making (Li et al., 2025). When drug responses can be predicted using organoids, patients may avoid exposure to ineffective drugs and their potential side effects (Ramezanpour et al., 2022). While patient-derived organoids offer significant individual benefits, distinct ethical issues remain.

First, when organoids are used to select or develop personalized treatments, maintaining the biological link between the patient and their organoid to return valuable results is crucial, yet protecting patient privacy is equally important. The use of organoids in personalized drug testing is associated with potential informational and physical harms, such as those arising from the return of unsolicited findings or privacy breaches (Muta and Nakanishi, 2025). Although organoids closely resemble the donor’s organ, they do not represent the entire organism. Therefore, the evidence they generate and the added clinical value of the technology cannot be directly extrapolated to the clinical setting (Lawlor et al., 2021). Thus, using organoid technology for personalized drug screening represents a departure from the standard translational pathway, where real-world evidence of a drug’s safety and efficacy is typically derived from large, comparable patient cohorts (Casiraghi and Remuzzi, 2025). This may blur the boundaries between research and clinical practice.

Additionally, the expectation of personal therapeutic benefit should not be considered a primary motivation for patients to participate in organoid research, as there is currently no guarantee that patients will receive compensation or access to new therapies developed using “their” organoids. The novel evidence generated from personalized drug screening also necessitates new reimbursement policies from insurers, who traditionally rely on safety and efficacy data from large-scale clinical trials (Cai et al., 2025).

#### 3.3.2 Human organoid transplantation

A second potential future clinical application is organ replacement therapy, where organoids are envisioned as a source of functional tissue for transplantation (Tabibzadeh and Morizane, 2024). For instance, liver organoids could potentially restore function in patients with metabolic liver disease. Preclinical animal studies suggest that liver organoids may be suitable for human transplantation and could 1 day become a less invasive alternative to organs from deceased donors (Farshbaf et al., 2024). Furthermore, combining organoids with gene-editing technologies holds promise for generating repaired, healthy organoids from patients with genetic defects for transplantation, though this raises its own ethical considerations.

As transplantable organoids move toward the clinic, their safety and efficacy must be rigorously tested following the traditional translational cascade—from basic research to animal studies, to first-in-human (FIH) trials, and finally to larger randomized controlled trials—each with appropriate ethical oversight (Bell et al., 2020). In human trials, risks should be minimized, and potential individual benefits and social value maximized (Pastore et al., 2025). Currently, evidence of potential benefit comes only from animal models and does not guarantee individual benefit for human patients (Wang et al., 2024). Transplantation of lab-grown organoids is a more invasive and complex procedure than traditional drug trials, representing a novel therapeutic concept. Given the associated uncertainties and lack of human evidence, such trials could expose participants to significant and potentially unreasonable risks (Shao et al., 2025).

Organoid transplantation trials might be ethically permissible if they ensure risks are minimized and benefits maximized, follow established clinical translation procedures, generate sufficient evidence to mitigate uncertainties, and guarantee that the trial design offers participants a realistic chance of medical benefit (Street et al., 2024). Several concrete suggestions have been proposed for organizing such trials responsibly (Zhang et al., 2024; Shinozawa et al., 2021):

1. Conduct combined safety and efficacy trials, rather than strictly phased trials (i.e., participants receive the intended therapeutic dose with efficacy as a primary endpoint).
2. Empower ethics committees to determine whether preclinical evidence is sufficiently compelling to justify potential individual benefit for participants in FIH transplantation trials.
3. Implement lifelong follow-up of participants.
4. Incorporate participants’ perspectives in the trial’s risk-benefit assessment, informed consent process, and patient selection.
5. Place strict emphasis on informed consent and, where applicable, assent.
6. Foster proactive interdisciplinary dialogue among scientists, policymakers, ethicists, the public, research participants, and clinicians.

Furthermore, organoid transplantation raises psychological and social issues. It may generate “soft impacts,” potentially affecting the recipient’s quality of life, their perception of their composite body (composed of their own tissue and the foreign organoid), and societal views on organ failure. For instance, the public might no longer view organ failure as invariably fatal, but rather as a condition for which a personalized replacement could be developed in a laboratory (Chen et al., 2025; Nikolaev et al., 2020).

#### 3.3.3 Clinical decision pathway example: organoid drug sensitivity guiding salvage therapy for advanced thyroid cancer

Consider a patient with locally advanced thyroid cancer for whom conventional treatments were limited due to high surgical risks. Patient-derived tumor organoids were constructed and subjected to high-throughput drug sensitivity testing, revealing high sensitivity to the targeted drug donafenib—a finding not entirely consistent with routine genetic testing. The clinical team adjusted the treatment plan accordingly, using donafenib as neoadjuvant therapy. This led to significant tumor shrinkage, creating an opportunity for curative surgery and ultimately validating the treatment’s efficacy. This case demonstrates that in “salvage settings” where standard treatments have failed or pose extreme risks, rigorously validated organoid drug sensitivity data can serve as key decision-making evidence (Evidence Level A). However, its application must follow a prudent pathway integrating sample quality control, technical validation, and dynamic clinical assessment.

Currently, organoid results should in most cases be regarded as crucial supplementary evidence rather than the sole basis for independent treatment decisions. Their role is highly dependent on the specific clinical scenario and the strength of the evidence. Clinicians should avoid basing treatment decisions solely on organoid data in the following situations:

Technical feasibility red line: organoid culture failure, sample contamination, or construction quality failing to meet preset laboratory quality control standards (e.g., low cell viability, inability to form three-dimensional structures).

Result reliability red line: organoid drug sensitivity results are inherently ambiguous or contradictory (e.g., all drugs show partial sensitivity/resistance), or the technology platform lacks rigorous clinical validation (e.g., its predictive sensitivity/specificity has not reached recognized thresholds in independent cohorts).

Clinical consistency red line: organoid results fundamentally conflict with other core clinical evidence for the patient (e.g., genetic testing indicates a classic sensitive mutation for a drug, but the organoid shows resistance). In such cases, the reliability of both the experiment and the genetic test should be re-evaluated before abandoning the standard treatment plan.

Legal and ethical foundation red line: the treatment plan based on organoid data falls completely outside current medical norms or regulations and has not undergone ethical review and informed consent (e.g., using an off-label drug combination identified by organoid screening without ethics committee approval and special patient consent).

To standardize the clinical translation of organoid technology, this study proposes specific operational requirements. For personalized drug screening, organoid platforms should achieve clear validation performance thresholds (e.g., predictive sensitivity ≥ 85%) prior to clinical use. A mandatory physician-patient communication checklist disclosing technical uncertainties should be established. Data governance must comply with human genetic resource regulations, and reimbursement models should explore evidence-based pathways. For organoid transplantation, a stepwise clinical translational framework is recommended, defining prerequisites for advancing from preclinical studies to human trials (e.g., completing systematic safety evaluations and establishing GMP-level production), specifying the minimum required data package (including CMC documentation and non-clinical safety data), and outlining persistent oversight mechanisms and explicit stopping rules for ethics committees (e.g., establishing an independent Data and Safety Monitoring Board). Furthermore, ethics committees need to enhance their review capabilities through expert augmentation and ongoing training to ensure effective implementation (Lei et al., 2026).

### 3.4 Data and privacy

Currently, the application of organoid technology in drug development is concentrated in basic research, drug discovery, and preclinical studies. As models that highly simulate human organs or tissues, organoids can replace or complement relevant animal experiments for *ex vivo* evaluation of drug efficacy and safety (Hong et al., 2024). When used for personalized treatment, a unique advantage is the ability to maintain a close biological link between the patient and their organoid to obtain valuable research results. However, this link simultaneously raises significant donor privacy concerns (Liu et al., 2024). Biological materials from donors, especially those with certain diseases, may contain sensitive personal health information. Balancing privacy protection with necessary information sharing is therefore critical (Yoshimura et al., 2023). Unauthorized disclosure of experimental findings could violate donor privacy or cause harm, making the effective and ethical utilization of this information a significant challenge (Inagaki et al., 2025).

### 3.5 Bioethical controversies

#### 3.5.1 Ethical issues specific to organoid subtypes

Brain organoids have been successfully used to model conditions such as Zika virus-induced microcephaly, idiopathic autism, and schizophrenia (Birtele et al., 2025). They have advanced our understanding of brain tumors, neurodevelopment, and neurodegeneration. Future applications might even include repairing brain circuits after injury or stroke (Jeziorski et al., 2023). Furthermore, “assembloids”—brain organoids connected to other organoids—are under development to study inter-organ communication, such as the gut-brain axis (Hartung et al., 2024).

Brain organoid transplantation involves three scenarios, each raising profound ethical questions: 1) Transplantation into non-human animals, creating chimeras; 2) Connection with non-biological entities like AI or robots, creating hybrids; 3) Transplantation into human individuals. Since cognitive abilities, consciousness, and identity are closely linked to the brain (Giandomenico et al., 2017), chimeras or hybrids receiving human brain organoids may develop human-like attributes. This raises concerns about endangering human dignity, the moral anthropomorphization of animals or machines, and the blurring of boundaries between humans, animals, and technology (Lavazza, 2021a).

A core ethical issue is whether brain organoids could develop consciousness, especially given their ability to form neural networks and exhibit spontaneous electrical activity *in vitro* (Stankovic and Colak, 2022). If they possess consciousness, researchers might have ethical obligations toward them as entities with moral interests, potentially rendering certain experiments unethical. Conversely, some argue that consciousness is impossible without interaction with a social environment (Kataoka et al., 2025). Viewing organoids as beings with moral status also raises questions about donor consent and control. Can donors withdraw consent if “their” organoid develops consciousness? Further research is needed to understand how to detect and possibly prevent consciousness in brain organoids (Lavazza, 2021b).

Consequently, some scholars advocate for strict ethical oversight of brain organoid research, potentially by specialized review boards (Hostiuc et al., 2019), including minimizing the number of brain organoids used. Others call for developing special ethical guidelines or international regulatory frameworks to ensure the welfare of brain organoids in clinical research (Hyun et al., 2020).

Gastruloids, a subtype replicating early developmental processes rather than organs, spark intense ethical debate. Controversies focus on the ethical status of such embryo models, the boundary between them and natural embryos, and the mismatch between rapid technological progress and lagging regulatory policies (Sawai et al., 2019). This triggers legal challenges, such as whether the “14-day rule” for human embryo research should apply (Giandomenico et al., 2021). Globally, ethical norms for embryo model research are relatively insufficient, often relying on outdated regulations for human embryos and stem cells that have not adapted to this specific, fast-evolving field.

The advancement of organoid technology extends beyond *in vitro* culture to implantation into animals, creating “chimeras” to validate function or model diseases. This cross-species fusion raises significant ethical concerns, especially with human brain organoids (Fiorenzano et al., 2021). For instance, in 2022, US scientists transplanted human stem cell-derived brain-like structures into rats, which then sent and received signals in response to environmental cues (Zhou et al., 2025a). While no conclusive evidence suggests these animals acquired human-like consciousness, such experiments have sparked profound discussions on the blurring of human-animal boundaries, the moral status of animals, and the generation of consciousness.

These controversies fuse ethical concerns from brain organoid and chimera research (Munsie et al., 2017), touching not only on scientific boundaries but also on deep moral and philosophical questions regarding human responsibility, animal welfare, the dignity of life, and human dignity itself.

### 3.6 Animal ethics

Chimeras are defined as organisms composed of cells from two or more species. The transplantation of organoids into animals is primarily aimed at enhancing their vascularization, thereby enabling them to grow *in vitro* beyond their otherwise achievable size limit and producing more representative models of human development and disease (National Academies of Sciences et al., 2021; Heidari et al., 2023). Despite these advantages, the concept of creating chimeras by transplanting brain organoids into animals raises ethical concerns, particularly regarding the “humanization” of the brain. In experiments where brain organoids have been transplanted into rodents, the grafts have demonstrated advanced neural differentiation, gliogenesis, microglial integration, and axonal growth into multiple host brain regions (Gaillard et al., 2025). Imaging studies have even revealed functional neural networks and vasculature within the transplanted organoids (Menon et al., 2023). A significant concern is that integrating human cells into an animal’s central nervous system could potentially lead to “human-like consciousness” or “self-awareness,” endowing the chimera with a morally ambiguous status (Faltus et al., 2023). Given the presence of human genetic material, some argue that chimeras may possess greater intrinsic interests or are entitled to greater respect compared to other animals. Consequently, there are calls for research involving chimeras to adhere to stricter guidelines and ethical oversight, with some suggesting that laboratory mice displaying advanced cognitive abilities should not be euthanized at the end of a study or should receive special consideration (Gabriel et al., 2024).

To safeguard animal welfare, some researchers propose that chimeras may require specific forms of management (Lee et al., 2025) or even be granted a degree of agency in decisions about their participation in research. Furthermore, another ethical issue raised is that human dignity could be undermined if animals acquire cognitive or mental capacities traditionally considered unique to humans (Lewis-Israeli et al., 2021). Future research should focus on determining which types of brain enhancement in animals are morally significant, how to identify such enhancements when they occur, and how to prevent them. Proposed measures include limiting the number of human stem cells transplanted, closely monitoring animal behavior and physiological changes, and employing mirror tests (i.e., observing an animal’s reaction to its own reflection) to assess self-awareness. However, some scholars contend that under current laboratory conditions, it is highly improbable for animals to develop human-like consciousness, and therefore, this concern does not present a serious ethical barrier to progress in organoid research (Shannon et al., 2024).

Another issue pertains to donor consent: donors may disapprove of using organoids (neural or otherwise) derived from their biological tissue to create chimeras and might refuse or withdraw consent. It has been suggested that future informed consent procedures should explicitly inform donors of such potential applications. Additionally, unresolved questions remain regarding the legal ownership of enhanced or potentially conscious chimeras. Finally, transplanting human gonadal organoids (e.g., testicular or ovarian organoids) into animals raises profound ethical concerns about the potential for cross-species reproduction between humans and non-human organisms—an outcome that experts agree should be strictly avoided (Gjorevs et al., 2022).

### 3.7 Benefit-sharing

#### 3.7.1 Benefit-sharing principles in commercialization

To safeguard donor rights and interests, direct monetary transactions are typically avoided to prevent organ commodification. Instead, non-monetary compensation is provided through means such as medical benefit-sharing (e.g., priority access to derived therapies) or granting rights to participate in research. Regarding intellectual property ownership, it is essential to clearly stipulate in the informed consent document whether donors hold partial rights to technologies derived from their tissue, such as entitlements to patent royalties. When academic institutions collaborate with industry, benefit allocation—such as patent licensing fees and equity distribution—should be explicitly defined through technology transfer agreements to prevent the inappropriate monopolization of academic achievements (Harkin et al., 2024). If research relies on public funding or public resources (e.g., hospital biobanks), a portion of the commercialization profits should be returned to public health initiatives, such as funding for rare disease research. Furthermore, a tiered benefit model can be employed for benefit sharing. This encompasses direct benefits for donors, such as free or low-cost organoid-derived therapies (e.g., personalized drug screening services), as well as indirect benefits achieved by reducing healthcare costs through technology dissemination or by supporting targeted health projects in source communities (e.g., screening programs for specific diseases). Biopiracy practices, such as collecting samples from low-income countries for commercialization in developed countries, must be avoided. Adherence to international norms like the Nagoya Protocol is required to ensure fair benefit-sharing for resource providers (Vistoso Monreal et al., 2024). Concurrently, transparency mechanisms need to be established. These should include a public benefit flow tracking system and the regular disclosure of commercialization progress and benefit distribution to stakeholders, including donors, the public, and funding agencies. Additionally, standardized contract templates must be developed, along with standardized protocol templates for informed consent and benefit sharing, to reduce information asymmetry and power imbalance (Li Z. et al., 2022).

The ethical framework for organoids needs to address the structural inequalities between high-income countries (HICs) and low- and middle-income countries (LMICs) from multiple dimensions. This can be achieved through five key measures: First, establish a fair resource and data sharing mechanism. HIC-led research institutions must open access to standardized organoid sample banks, technical protocols, and core databases. They must clarify the ethical boundaries of cross-border sample use, prohibit exclusive commercial exploitation, and ensure LMIC researchers’ core participation rights along with the territorial retention and sharing rights of research data. Second, build an inclusive benefit distribution system. This involves mandating the tiered sharing of commercialization gains from organoid research, allocating a portion of profits to medical infrastructure construction and local scientific research in LMICs, prioritizing their access to derived therapies, and setting up special funds to provide non-material reciprocal compensation to resolve the “burden-benefit mismatch.” Third, promote localized adaptation and international coordination of ethical supervision. This includes assisting LMICs in establishing context-specific ethical review systems that incorporate cultural sensitivity indicators, building a cross-border supervision alliance to unify core ethical standards, and establishing mechanisms for technical support and qualification recognition. Fourth, strengthen the scientific research and ethical capacity of LMICs. This requires incorporating technology transfer and talent training into mandatory requirements for international cooperation and setting up special grants to support organoid research in LMICs that targets local diseases, thereby helping to reverse the imbalance in scientific research discourse power. Fifth, improve the inclusive design of informed consent. This involves adopting multilingual and plain-language consent documents in response to language and cognitive disparities, introducing community collective decision-making mechanisms, safeguarding donors’ control over samples through dynamic consent tools, and repairing the trust deficit caused by historical medical exploitation.

In the cross-border data and biological resource governance of organoid biobanks, it is essential to clarify the regulatory logic of the two major legal frameworks and the applicable pathways of the core principles of the Nagoya Protocol. From the perspective of data privacy and cross-border transfer regulations, the European Union’s General Data Protection Regulation (GDPR) establishes security boundaries for the cross-border flow of donor personal health information in organoid biobanks. It does this through provisions such as tiered privacy protection, “adequacy decisions” for cross-border data transfers, and robust data subject rights. This requires research institutions to undergo compliance review and establish robust privacy protection mechanisms before engaging in international data sharing. In the United States, the Health Insurance Portability and Accountability Act (HIPAA) applies the “minimum necessary” principle for data use and employs standard data confidentiality agreements to regulate the entire process of storing, accessing, and applying donor health information across borders within biobanks, ensuring compliance and security in data usage.

Regarding the utilization of derived biological resources such as organoids in international cooperation, the Nagoya Protocol’s principle of “prior informed consent” requires research entities to obtain explicit authorization from management authorities and relevant communities in the sample source location before accessing organoid research materials, thereby completing full informed notification and authorization procedures. Furthermore, its “benefit-sharing” principle defines the allocation rules for research outcomes, emphasizing that health services, scientific achievements, and economic benefits generated from organoid research should be reasonably shared with the sample-providing communities. This helps prevent imbalances of interest in the cross-border utilization of biological resources.

### 3.8 Oversight and compliance

Researchers propose that ethical review for organoid research should involve a two-tiered system: First Tier: Routine Review: or research that does not raise novel ethical or regulatory issues, approval can be obtained through the standard review and approval process conducted by an Institutional Science and Technology (Ethics) Review Committee. This tier includes: *in vitro* studies generating human brain organoids lacking phenomenal consciousness (such as the capacity to feel pain) from pre-implantation human embryos, patient/individual-derived induced pluripotent stem cells, or somatic cells; and chimera research that does not significantly enhance the sensation, cognition, or consciousness levels of the host animals. Existing regulatory frameworks concerning chimera research and animal welfare apply to these activities, obviating the need for new legislation or specific ethical guidelines (Tabibzadeh et al., 2023). However, it remains crucial to assess whether humane care and attention, commensurate with their potential levels of consciousness (e.g., a capacity to feel pain), are provided to such human brain organoids and chimeras (Taelman et al., 2022).

Second Tier: Expert Re-review: For human brain organoid research that may pose significant ethical risks or challenges, it must first undergo a preliminary review by the Institutional Science and Technology Ethics (Review) Committee. The institution must then submit the proposal to the relevant local or sectoral authority to organize an expert re-review, and the research may proceed only upon receiving approval (Davies et al., 2024). This tier encompasses: *in vitro* studies generating human brain organoids possessing phenomenal consciousness (e.g., pain sensation) or higher levels of consciousness; chimera research that significantly enhances the sensation, cognition, or consciousness levels of non-primate host animals; research on human brain organoid hybrids that may develop phenomenal consciousness (e.g., pain sensation); and research involving the transplantation of human brain organoids into human individuals (Ranganathan et al., 2020).

Given that each research protocol entails different potential benefits and risks, reviews should be conducted on a case-by-case basis. The research mentioned above should be pursued cautiously and under the following stipulated conditions:

Allowing the introduction of harmful genetic alterations via gene editing or other means in specific regions of human brain organoids, while prohibiting such alterations in the entire human brain organoid.

Administering sedatives to human brain organoid grafts during invasive or destructive experiments. Closely monitoring the physical and behavioral changes in human brain organoid chimeras (especially those involving non-human primates) and hybrids (Wang and Fu, 2024). If chimeras or hybrids are found to possess phenomenal consciousness (e.g., pain sensation), experiments may continue only under fully justified circumstances and with due respect. If chimeras or hybrids are found to possess human-like self-awareness, a structured approach is required: First, “human-like self-awareness” should be defined as a scientific hypothesis and risk assessment criterion based on neuroscientific evidence—such as specific whole-brain electrical activity patterns and cognitive-behavioral responses to mirror self-recognition—rather than a state that can be easily determined. Second, continuous, pre-set multi-index monitoring should be implemented for such high-risk research, integrating dimensions like neural activity complexity and behavioral spectroscopy analysis. Third, it must be clarified that under current scientific understanding, discussing the “consent” of chimeras has no practical operability. Therefore, the core ethical requirement is to establish strict upper-limit rules (e.g., strictly prohibiting reproductive capacity or development beyond a specific stage) while setting clear risk thresholds. Once monitoring detects signals approaching these thresholds (such as abnormally complex integrated neural activity), an immediate suspension of the experiment and a mandatory ethical review must be triggered, thereby translating abstract ethical principles into concrete, executable steps within the review process.

Human brain organoids, chimeras, and hybrids whose research or experimentation is terminated should be placed in a suitable environment. We propose establishing a specially designed protocol for determining the “end of life” and subsequent disposal of brain organoids or highly integrated neural chimeras upon research termination. This protocol should not directly adopt human brain death criteria but instead set objective determination indicators based on their neural activity characteristics—for example, after withdrawing artificial life-support systems, continuously monitoring until global field potential activity completely ceases. This process aims to establish rigorous, actionable, and ethically sound criteria for defining their definitive end-of-life status.

“Ethical Governance” is the ultimate goal of science and technology ethics governance. It is recommended to promptly establish a research consortium encompassing neuroscientists, stem cell biologists, bioethicists, ethologists, experts in artificial intelligence and robotics, research funders, and the public (including donors and patients). There is an urgent need for in-depth, interdisciplinary dialogue among consortium members to comprehensively study and understand the scientific basis and social value of emerging technologies. This will ensure the real-time identification of adverse effects, proactive addressing of ethical challenges, and establishment of necessary legal boundaries from within the consortium. Targeted ethical governance frameworks and specific norms should be developed to help consortium members enhance their awareness and capacity for ethical reflection, optimize research design, and track the entire implementation process, thereby better predicting and controlling the ethical risks of research and maximizing the potential scientific value and social benefits of related research (Mashouf et al., 2024).

#### 3.8.1 An “extension” of stem cell ethics

The ethical issues surrounding organoids can essentially be positioned as a critical extension and concrete application of stem cell ethics and the broader ethical framework for human research. We systematically map the proposed eight ethical review dimensions to existing authoritative principles and guidelines: Source legitimacy and informed consent are grounded in the principle of respect for persons from the Belmont Report and the core autonomy principle of the Declaration of Helsinki, directly echoing the stringent requirements for donor consent in ISSCR guidelines (International Society for Stem Cell Research ISSCR Standards Initiative, 2023). The dimensions of scope of use restrictions and animal ethics, particularly constraints on brain organoids, embryo models, and human-animal chimeras, refine and extend the relevant risk management clauses in ISSCR guidelines, aiming to fill gaps in defining “consciousness” thresholds and quantitative standards for cross-species integration (International Society for Stem Cell Research ISSCR, 2021). The dimensions of data and privacy, benefit-sharing, and oversight and compliance represent the concrete practice of regulations like the General Data Protection Regulation (GDPR), biobank ethical frameworks, and research integrity norms within the novel and dynamic field of organoids (National Health Commission of the People’s Republic of China and Ministry of Science and Technology of the People’s Republic of China, 2024). This mapping is not a mere application but reveals the necessary interpretation and adaptation of existing principles when confronted with the “quasi-life” attributes, long-term cultivation potential, and commercialization prospects of organoids.

Building on this, the preliminary practices in different jurisdictions present a divergent landscape: the European Union tends toward cautious regulation within existing strict biotechnology and data protection frameworks (e.g., the Advanced Therapy Medicinal Products Regulation, GDPR); the United States relies more on the flexibility of the Institutional Review Board system and specific restrictions in NIH guidelines, presenting a decentralized character; in Asia, taking China’s newly released Ethical Guidelines for Human-Derived Organoid Research (2024) as an example, efforts are underway to establish more targeted national-level ethical review checklists and negative list systems. This comparative analysis highlights the urgency of global governance coordination (Zhou and Han, 2025).

Furthermore, to address unique challenges, current guidelines urgently require supplementation with specific content. For brain organoids, ISSCR and national guidelines need to define monitoring thresholds for electrophysiological activity or other biomarkers and establish tiered review procedures and disposal standards accordingly. For chimera research, it is necessary to move beyond principled prohibitions by defining acceptable upper limits for the colonization proportion, spatial distribution, and functional integration degree of human cells in animal hosts (especially the central nervous system). These specific parameters must be incorporated as mandatory disclosure and assessment requirements in informed consent and ethical review. This study aims to provide a theoretical foundation and actionable guidance for constructing a coherent yet flexible governance system capable of responding to the rapid evolution of the technology.

## 4 Discussion

Through a systematic review of the ethical issues in organoid technology, this study proposes, for the first time, a comprehensive ethical review framework encompassing eight dimensions: source legitimacy, informed consent, scope of use restrictions, data and privacy, bioethical controversies, animal ethics, benefit sharing, and oversight and compliance. In contrast to the prevailing tendency in existing literature to address specific issues in isolation—such as an excessive focus on the potential consciousness of brain organoids—the unique contribution of this framework lies in integrating scattered ethical concerns into a logically coherent and actionable structured system. It particularly emphasizes the ethical risks associated with non-neural organoids (e.g., gonadal organoids) and the multi-tiered regulatory needs ranging from individual consent to global governance. We argue that the moral governance of organoid technology must move beyond principled debates and be translated into concrete review checklists, phased regulatory pathways, and executable benefit-sharing mechanisms.

A systematic review focusing on the key points for the ethical review of organoids has not yet been identified. This review encompasses all ethical issues mentioned in the literature on organoid technology, aiming to provide assistance during the clinical review of organoid projects.

Concurrently, other subtypes of organoids, including gonadal organoids, and the ethical debates surrounding their potential clinical applications, have been somewhat overlooked in the current literature. As gonadal organoid technology could increase the possibility of reproduction for both males and females, as well as for other life forms including human-animal chimeras, it warrants serious ethical consideration (Lin et al., 2025). We encourage future research to investigate more deeply the ethical issues associated with gonadal organoids. Furthermore, the governance framework for the potential future applications of organoid technology is currently inadequate. As these technologies may soon be ready for clinical trials and clinical implementation, they raise very real and practical ethical questions that urgently demand clarification, particularly regarding the ethics surrounding the use of organoids for personalized medicine.

In summary, organoid research is advancing rapidly, and it is crucial to continue monitoring developments related to organoid technology, which may necessitate new and more detailed ethical analyses. The purpose of such monitoring is to address potential opportunities and risks without leading to unnecessary restrictions on organoid research. Currently, the ethical literature on organoids appears disproportionately focused on specific subtypes—particularly brain organoids, human-animal chimeras, and gastruloids. This has resulted in inaccurate and incomplete descriptions of potential future organoid applications, which may unduly influence public perception and ethical debate. More ethical research is needed in areas where clinical applications are closer to fruition (Zhou et al., 2025a). For instance, there is relatively limited ethical discussion on the use of organoids in human transplantation and on the impact of such applications on patients, healthcare organizations, and society as a whole. Additionally, there is a need for empirical studies on donor and public perspectives in various settings to adequately address public concerns about organoids.

From an ethical perspective, the ethical review of organoids is a “complex entity.” Firstly, while organoids are a technological achievement, they are intimately linked to donors through issues like privacy, thus forming an inseparable ethical connection with them. Future research needs to further explore how to overcome these barriers to achieve effective and operable ethical governance for organoids (Zhou et al., 2025b).

**FIGURE 3:**
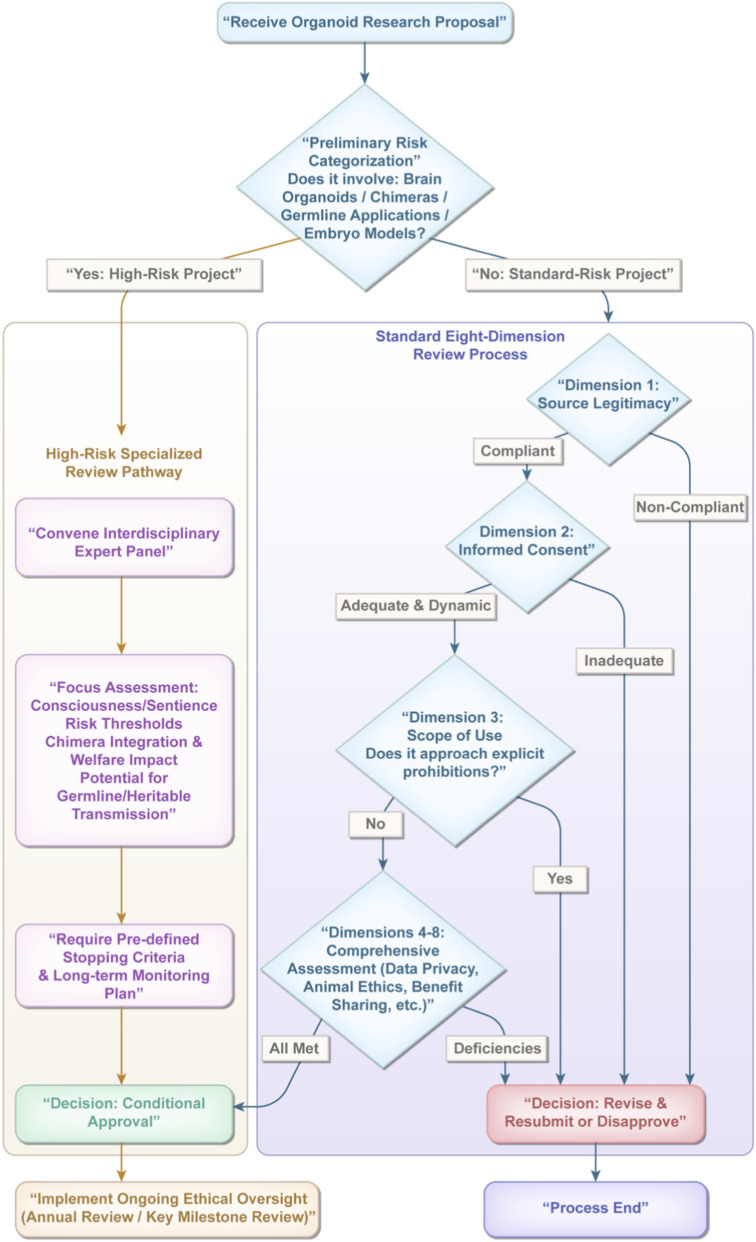
Ethics committee decision tree for organoid ethical review.

First and foremost, strengthening interdisciplinary collaboration among biomedical experts, ethicists, legal experts, and sociologists is crucial to ensure comprehensive consideration from scientific, ethical, and legal perspectives. Furthermore, given the specificity of the organoid field, refining the informed consent process is fundamental for protecting donor rights and privacy. This requires providing donors with clear and detailed information, ensuring they have a complete understanding and decision-making authority over the use of their biological materials. Simultaneously, establishing internationally harmonized ethical guidelines, enhancing societal awareness of this field through public education and engagement, and establishing dedicated ethics review committees within research institutions to conduct professional ethical reviews of organoid research are all essential steps to ensure adherence to ethical and scientific standards (Zhou et al., 2025c) (Figure 3).

Building upon this framework, this paper further clarifies several critical issues requiring urgent resolution. In the short term, the priority for regulatory bodies and ethics committees is to establish minimum ethical standards for organoid research—particularly concerning informed consent templates and biobank access—and to initiate interdisciplinary efforts aimed at defining consciousness thresholds for brain organoids (Liu et al., 2025). In the medium term, the focus should shift to developing model agreements for cross-border data sharing and benefit-sharing, as well as creating risk-stratified regulatory tools applicable to different types of organoids (Pryzhkova et al., 2022). In the long term, it is essential to promote international dialogue to establish a coherent global governance framework, prevent “ethics dumping,” and address the fundamental bioethical challenges that organoid technology may present.

To advance this agenda, we propose the following concrete action recommendations: For Ethics Review Committees: Immediately adopt or refer to the eight dimensions of this framework as a review checklist. Require specialized review for high-risk studies (e.g., those involving brain organoids or highly integrated chimeras) and mandate the evaluation of benefit-sharing plans. For Researchers: Integrate ethical considerations at the project design stage. Employ dynamic informed consent processes, proactively disclose technical pathways and potential applications, and actively participate in developing field-specific best practice guidelines. For Policymakers: Fund prospective studies on the ethical, legal, and social implications (ELSI) of organoid technology (Kawai et al., 2024). Expedite the issuance of national guidelines for organoid research and support the establishment of cross-institutional ethics advisory and oversight networks (Ye et al., 2024). Through this structured framework, clear priorities, and concrete, coordinated actions from multiple stakeholders, we can guide the responsible development of organoid technology. This approach will help ensure it truly benefits human health while safeguarding ethical boundaries and upholding principles of fairness and justice to the greatest extent possible.

## Glossary

Organoid: A miniature, lab-grown tissue that forms under 3D culture conditions. It starts from stem cells or organ-specific progenitor cells, which self-organize and mature to mimic an actual organ’s architecture, cellular makeup, and some of its functions. It acts as a vital research model that sits between flat, 2D cell cultures and whole living organs.
Organoid Co-culture model: An experimental setup where scientists grow different types of organoids together, or combine organoids with other cell types (like immune cells or blood vessel cells), in the same dish. This allows researchers to model how organs interact or to recreate specific disease environments. For example, co-culturing brain and spinal cord organoids can help study nerve connections, while growing tumor organoids with immune cell organoids can model how cancers interact with the immune system to test new therapies.
Organoid Technology: The entire toolkit of methods for creating, growing, maintaining, analyzing, and genetically modifying organoids. It also covers their wide-ranging uses in modeling diseases, testing drugs and toxins, studying development, and working towards regenerative medicine treatments. The core goal is to recreate the intricate structure and function of living tissues in the lab.
Brain organoid (cerebral organoid): A specific type of organoid grown in the lab from human pluripotent stem cells. These organoids develop some of the distinct structures (like layered cortex) and cell types (such as neurons and astrocytes) found in specific brain regions. They are a key model for understanding human brain development, neurological diseases, and the basis of cognition. Their complexity also raises serious ethical questions about potential consciousness and how they should be regarded.
Organoid assembly/assembled organoid system: Using bioengineering tools—like 3D bioprinting or microfluidic chips—to carefully combine multiple organoids or organoid modules with supporting elements (e.g., blood vessel networks or scaffold materials). The aim is to build more sophisticated, interconnected multi-organ systems or “body-on-a-chip” models that better reflect human physiology.
Gastric organoid (stomach organoid): An organoid that replicates the structure and function of the stomach’s inner lining. It typically contains key stomach cell types like acid-producing parietal cells, enzyme-secreting chief cells, and hormone-releasing endocrine cells. This model is invaluable for studying normal stomach function, diseases (like gastritis and stomach cancer), and infections such as those caused by *H. pylori*.
Chimera: In organoid research, this usually means human-animal chimeras. These are mixed organisms created by introducing human cells (which can include stem cells or organoids) into early animal embryos or adult animals, where they grow and integrate. This research is important for studying human development, disease, and the potential for growing human organs in animals, but it comes with major ethical concerns about animal welfare, human dignity, and the blurring of species boundaries, including potential effects on the animal’s mind.
Hybrid: This term is sometimes confused with “chimera,” but it technically refers to something created at the single-cell level by fusing genetic material from different species (e.g., creating hybridoma cells for antibody production). It's less common in organoid discussions. The main ethical worry here centers on the mixing of genomes and crossing species lines.
Potential life/quasi-life attributes: An ethical and philosophical description applied to certain, highly complex organoids (like advanced brain or heart organoids). This is because they exhibit key features reminiscent of living things, such as self-organization, electrical activity, the ability to contract, or responses to stimuli. While not full organisms, they are more than just simple cell clumps, challenging the clear-cut line between what is considered alive and not alive.
Quasi-life entity: A term that follows from “quasi-life attributes.” It is used for organoids or engineered tissues that show significant signs of being “life-like.” The label highlights that in ethical discussions, they may warrant a different kind of consideration and respect than ordinary biological samples.
Phenomenal consciousness: A concept from philosophy and cognitive science that refers to the capacity for subjective experience—what it “feels like” to be that entity, to have sensations or feelings. In debates about organoid ethics, a central and challenging question is whether highly advanced brain organoids could ever develop even a rudimentary form of such inner experience. The answer is crucial for determining their moral standing.
Soft impact: The indirect, non-forceful way that organoid technology—through its images, ideas, and findings—shapes public understanding, cultural stories, social values, and policy discussions. For instance, seeing pictures of brain organoids can change how people think about “the brain” and “consciousness,” which in turn influences how society views the ethics and laws that should guide this research.
Moral status: Whether an entity, based on its own inherent features (like the ability to feel pain, consciousness, rationality, or self-direction), deserves moral consideration, has certain rights, or should not be used purely as a tool. A major focus of current debate is whether organoids (especially brain organoids) have moral status, or what conditions might grant them some degree of it.
Organoid transplantation: A potential future therapy where lab-grown organoids are implanted into patients to repair injured tissue, replace lost function, or restore organ activity. This is a major clinical goal of organoid and regenerative medicine research. Ethical evaluation of this approach must rigorously address safety, effectiveness, long-term risks, the source of the cells used, and fair access to the treatment.
Embryo model: A lab-created model, made from stem cells, that mimics the structure and biology of specific early stages of human embryo development (like the blastocyst or gastrulation stage). Examples are “blastoids.” These models offer a powerful new way to study early human development, infertility, and birth defects. However, because they can closely resemble real embryos, they trigger serious ethical and regulatory questions about their moral status, appropriate research limits (e.g., whether the “14-day rule” applies or needs updating), and how complete their development could potentially be.
